# The relationship of maternal hepatitis B e antigen and response to vaccination of infants born to women with chronic infection

**DOI:** 10.1186/s12884-023-05815-y

**Published:** 2023-07-15

**Authors:** Hongxiu Jiang, Chao Chen, Deping Yuan, Xiajun Ye, Yan Chen, Guorong Han, Guanlun Zhou, Yuhao Ju, Minkai Cao

**Affiliations:** 1 Department of Obstetrics and Gynecology, The Second Hospital of Nanjing, Nanjing University of Chinese Medicine, Nanjing, 210003 Jiangsu China; 2 Department of Pediatrics, The Second Hospital of Nanjing, Nanjing University of Chinese Medicine, Nanjing, 210003 Jiangsu China

**Keywords:** Follow-up, HBV marker, Hepatitis B virus, Hepatitis B virus DNA, Maternal hepatitis B e antigen, Immunization

## Abstract

**Background:**

The relationship of maternal HBeAg and infants’ response to hepatitis B vaccine remains controversial. This study aims to observe the dynamic changes in infant birth HBV markers and study the time-varying effects of maternal HBeAg on vaccination response of infants born to women with chronic HBV infection.

**Methods:**

3163 infants born to HBsAg positive mothers including 1737 with maternal HBeAg positive in group A and 1426 negative in group B were enrolled eventually. Demographic information and laboratory tests were collected at birth, 7-12th and 24th month. The dynamic changes of infant HBV markers and HBsAb titers at different time points were compared between the two groups.

**Results:**

The infant HBV markers at birth displayed different modes. During the follow-up, we observed a significant downward trend in the positive rates of HBsAg, HBeAg, HBeAb and HBcAb. The HBsAg of two groups switched to negative at 7–12 months and HBeAg in Group A became negative at 24 months. The HBsAb titers of the infants in the two groups were 576.91(192.8–1000.0) vs 719.67(208.1–1000.0) at 7–12 months (Z = -3.049, *P* = 0.002) and 783.5(227.8–1000.0) vs 891.4(234.0–1000.0) at 24 months (Z = -0.853, *P* = 0.394). High HBV DNA viral load (OR 1.260, 95% CI 1.139–1.395, *P* < 0.001) and maternal HBeAg level (OR 1.003, 95% CI 1.002–1.003, *P* < 0.001) were associated with the higher HBeAg positive rate of infants.

**Conclusions:**

Maternal HBeAg did affect the infants’ immune response to vaccination and reduce the anti-response at 7-12th month temporarily, but these influences were negligible by 24th months after birth, which proved that the maternal HBeAg would not induce immune tolerance of infants from a long-term perspective.

## Background

Hepatitis B virus (HBV) infection is a major public health problem worldwide, especially in China. Currently, mother-to-child transmission (MTCT) is one of the most common forms of HBV infection and often leads to infection chronicity. Factors known to increase MTCT risk include maternal hepatitis B e antigen (HBeAg) positivity, high maternal HBV load, and instrumental delivery of children [1–3]. These parameters are generally evaluated together to predict the risk of MTCT [4, 5].

HBeAg is a derivative of the hepatitis B core antigen (HBcAg), which is secreted into the host’s circulation during viral replication [6]. Several studies have found that fetal exposure to maternal HBeAg could cause partial tolerance of the neonatal immune system to HBV [7, 8]. However, other studies found that fetal exposure to maternal HBeAg did not inhibit the antibody response to the neonatal hepatitis B vaccine or induce immune tolerance to HBV [9]. Therefore, the relationship of maternal HBeAg and response to vaccination of infants remains controversial.

Although existing studies have provided some useful and important insights into the role of HBeAg in neonatal HBV immune tolerance, there are still some shortcomings. First, reports about the relationship between maternal HBeAg and infant HBsAb are relatively limited, usually measuring these proteins at only one time point and having no long-term follow-up beyond 12 months. No large-scale reports have illustrated a dynamic change in the HBsAb levels of infants over a relatively long period. Study of the time-varying effects of maternal HBeAg on the efficacy of infant immunization has also been neglected. Second, the sample sizes used in previous studies were not large enough to provide analysis results of good statistical power.

The Obstetrics Department of the Second Hospital of Nanjing specializes in catering to pregnant women with chronic HBV infection; therefore, we have abundant clinical participant sources. To further clarify the above issues, we enrolled mothers infected with HBV and analyzed the potential risk factors for infants with HBeAg positive at birth. Then, we divided infants into two groups according to maternal-derived HBeAg positivity and observed dynamic changes in the birth HBV markers between the groups over a long follow-up period (from birth to 24 months). We also analyzed the time-varying effects of maternal HBeAg on the vaccination response of infants born to mothers with chronic HBV infection during the follow-up.

## Methods

### Study design and population

A study was conducted to observe dynamic changes in infant birth HBV markers and to assess the time-varying effects of maternal HBeAg on the vaccination response of the infants. Mothers who underwent prenatal examination and delivered at the Department of Gynecology and Obstetrics, The Second Hospital of Nanjing, Nanjing University of Chinese Medicine, were included in this study. They volunteered for the outpatient follow-up in clinical routine to confirm the infants’ immunization effects. All the infants born to mothers infected with HBV in our hospital receive HBV markers and HBV DNA assessments at birth. All infants enrolled were born between January 2013 and December 2018. Data were obtained from our database which was gathered previously and anonymized, with no direct participation of patients. Thus, informed consent was exempted.

The inclusion criteria were as follows: mothers infected with HBV; infants born to mothers with HBsAg positivity for more than 6 months, birth weight ≥ 1500 g, and follow-up to at least seven months. The exclusion criteria were as follows: mothers co-infected with hepatitis A, C, D, E, syphilis or human immunodeficiency virus (HIV); combined use of immune modulators, steroids, or cytotoxic drugs; infants with birth defects; and incompletion or non-adherence to the recommended 0-1-6-month (within two hours after birth, in the first month, and the sixth month) schedule of hepatitis B vaccination. Mother-infant pairs fulfilling the inclusion and exclusion criteria were enrolled in our study.

All term infants were vaccinated with three 10-µg doses of the genetically engineered HBV vaccine (Amehanxin vaccine Ltd. Company, Dalian, China) according to the standard 0-1-6-month vaccination regimen. The first dosage of hepatitis B vaccine was injected within two hours after birth at the delivery room. In addition, they received a 100 international unit (IU) dose of hepatitis B immunoglobulin (HBIG) (Si Chuan Shuyang Ltd. Company, Chengdu, China) within two hours after birth. All preterm infants (born at < 37 weeks of pregnancy) or low birth weight infants (< 2500 g) were vaccinated with the genetically engineered HBV vaccine at a dose of 10 µg, according to the additional vaccination regimen (within two hours after birth and in the first, second, and seventh months). In addition, they received a 100 IU dose of HBIG within two hours after birth. Vaccination was delayed if an infant’s vital signs were unstable at birth.

### Neonatal standard of care and laboratory tests

The HBV markers and HBV DNA of infants were assessed at birth, and HBV marker assessment was repeated in months 7 to 12 and month 24 after delivery (HBV DNA test was added if HBsAg is positive, and blood samples were collected from the femoral vein). Serum HBV DNA levels were determined through quantitative fluorescence polymerase chain reaction (PCR) (Shanghai Kehua Bioengineering Co., Ltd., Shanghai, China), which has a detection range of 5 × 10^2^–1 × 10^8^ IU/ml. Levels of serum HBV markers, including HBsAg, HBsAb, HBeAg, HBeAb, and HBcAb, were determined by the Abbott i2000 (Abbott Diagnostics, Abbott Park, IL, USA).

### Efficacy assessments

Firstly, we used binary univariate and multiple regression analysis to detect the potential risk factors of HBeAg positive rate of infants born to HBeAg positive mothers. Secondly, we divided the infants into the maternal derived HBeAg ( +) group (A group) and the maternal derived HBeAg (-) group (B group) according to the infants’ HBeAg status at birth and compared their HBsAb levels (index of immunization response) and the time-varying effects of maternal HBeAg on infants’ response to hepatitis B vaccines between the two groups. Thirdly, we observed the birth HBV marker expression of infants as well as the dynamic changes in the two groups.

### Definitions relevant to the evaluation of hepatitis B vaccination status in infants

Mother-to-child transmission was defined as detectable levels of HBV DNA or HBsAg positivity in peripheral serum samples of infants at birth and up to at least 6 months after birth [10, 11]. Immunization success in infants was defined as HBsAg negativity and an HBsAb level of ≥ 10 mIU/ml after completion of the standard vaccination regimen at the age of 7 months. Low immunization response was defined as HBsAg negativity and an HBsAb level of < 100 mIU/ml. Medium–high immunization response was defined as HBsAg negativity and an HBsAb level of ≥ 100 mIU/ml. No response to immunization was defined as HBsAg negativity and an HBsAb level of < 10 mIU/ml [12].

### Statistical analysis

The SPSS 23.0 software (SPSS, Inc., Chicago, IL) was used for statistical analysis. Normally distributed continuous variables were expressed as means ± standard deviation (SD) and tested by t-test while skewed variables presented as medians and interquartile ranges (IQR) and tested by nonparametric test. Frequencies and percentages were used to assess categorical variables. The chi-squared test or Kruskal–Wallis test was used to compare data. Binary univariate and multivariate regression analysis was used to reveal the risk and protective factors. Spearman’s correlation coefficient was used to determine the correlations between variables. All tests were two-tailed, with a 95% confidence interval, and statistical significance was considered at *P* < 0.05.

## Results

### Study participants and basic characteristics

**Fig. 1:**
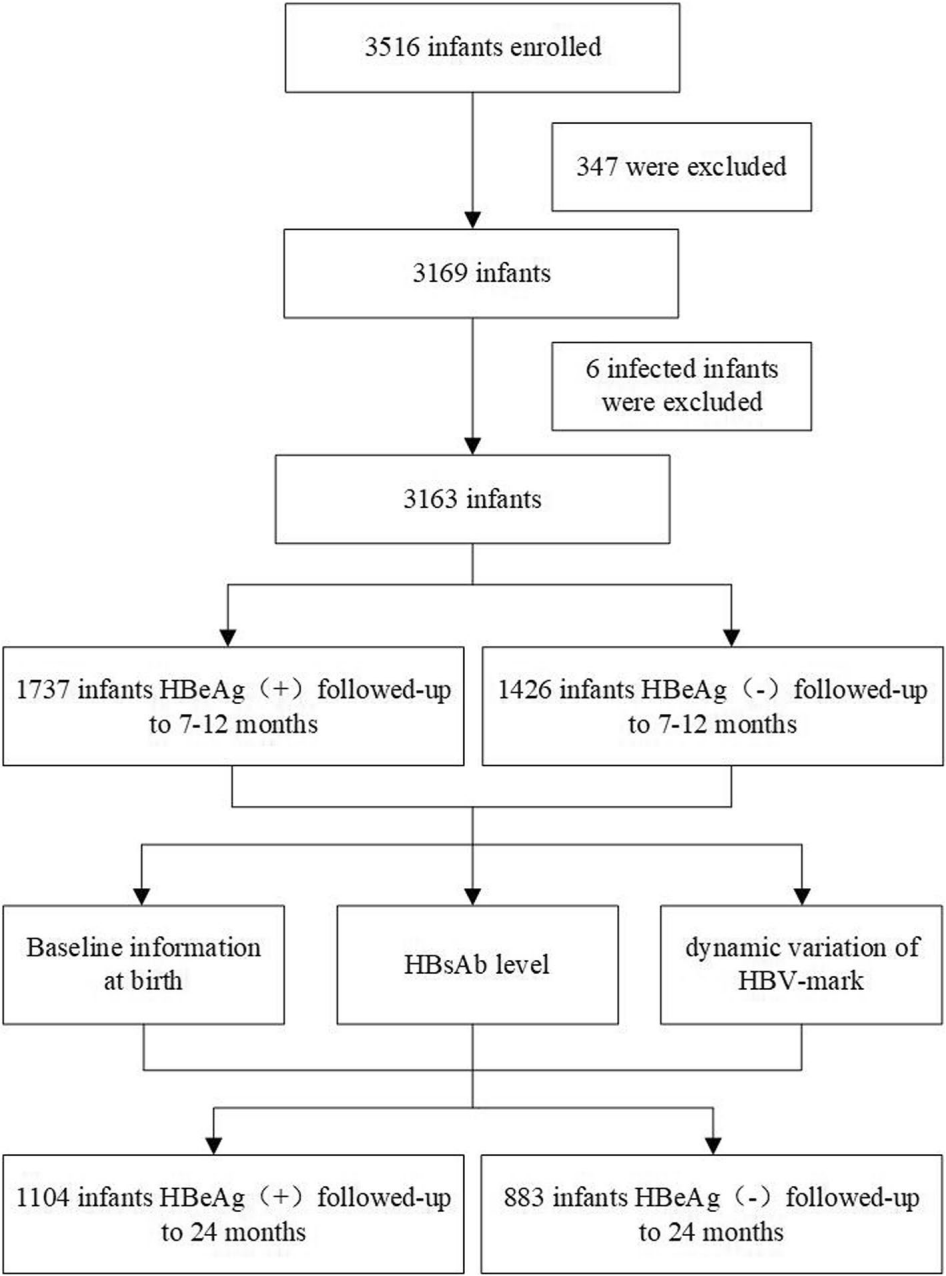
Flowchart of the study design

3516 mother-infant pairs were enrolled, 347 with incomplete data or failed to meet the inclusion criteria and 6 infants who had MTCT were excluded. 3163 mothers were enrolled including 1926 with HBeAg positive and 1237 HBeAg negative. The remaining 3163 infants were divided into two groups according to HBeAg positivity at birth: 1737 infants were present in group A (HBeAg positive group) and 1426 infants were present in group B (HBeAg negative group). All infants were singleton and followed up to at least 7–12 months. Among these, 1104/1737 infants in group A and 883/1426 infants in group B were followed up to 24 months. Our study design is shown in Fig. 1. The basic characteristics are presented in Table 1. Variables were compared between the two groups.

**Table 1:** Demographic features and clinical basal characteristics of infants

	A group (*n* = 1737)	B group (*n* = 1426)	Z or χ2	*P* value
Gestation week, median (range)	39.3(38.4–40.1)	39.1 (38.4–40.0)	-0.991	0.322
Preterm rate, n (%)	88(5.1)	70(4.9)	0.045	0.831
Male infant, n (%)	924(53.2)	748(52.5)	0.132	0.717
Infants of low-birth weight, n (%)	30(1.7)	32(2.2)	1.090	0.296
Birth weight (g), median (range)	3310(3060–3598)	3350(3080–3610)	-1.952	0.051
Birth height (cm), median (range)	49(49–50)	49(49–50)	-1.001	0.317
Infant deformity, n (%)	2(0.1)	2(0.1)	0.038	0.845
Asphyxia neonatorum, n (%)	10(0.6)	14(1.0)	1.715	0.190
1 min Apgar score, median (range)	10(10–10)	10(10–10)	-1.194	0.233
5 min Apgar score, median (range)	10(10–10)	10(10–10)	-0.262	0.793

### Expression modes of HBV markers at birth and dynamic changes from birth to 24 months

In our study, HBV markers at birth displayed different expression modes between the two groups and presented dynamic changes from birth to month 24 (Table 2). At birth, the HBsAg positivity rate in group A was 17.44% (303/1737), which was statistically higher than the HBsAg positivity rate of 4.56% (65/1426) in group B (χ^2^ = 125.224, *P* < 0.001). The HBsAg positivity at birth in the two groups and HBeAg positivity in group A were all temporary and switched to negativity during the 7-12 month follow-up. HBV markers, such as HBeAg, HBeAb, and HBcAb, can all be transmitted from mothers to infants. The expression modes of infants between the two groups were therefore different due to the difference in the mothers’ HBV markers.

**Table 2:** The expression modes of HBV markers at birth and their dynamic changes from birth to 24 months for infants who did not have HBV infection

	HBV marker	At birth (*n* = 1737/1426)	7–12 months (*n* = 1737/1426)	24 months (*n* = 1104/883)
Infants in A group	HBsAg ( +), n%	303(17.4)	0(0.0)	0(0.0)
	HBsAb ( +), n%	0 (0.0)	1734(99.8)	1092(99.0)
	HBeAg ( +), n%	1737(100.0)	223(12.8)	0(0.0)
	HBeAb ( +), n%	22(1.3)	12(0.7)	4(0.4)
	HBcAb ( +), n%	1715(98.7)	952(54.8)	341(30.9)
Infants in B group	HBsAg ( +), n%	65(4.6)	0(0.0)	0(0.0)
	HBsAb ( +), n%	0 (0.0)	1424(99.9)	870(98.5)
	HBeAg ( +), n%	0(0.0)	0(0.0)	0(0.0)
	HBeAb ( +), n%	1151(80.6)	856(60.0)	98(11.1)
	HBcAb ( +), n%	1423(99.8)	768(53.9)	271(30.7)

Abbreviation: *HBV* Hepatitis B virus, *HBsAg* Hepatitis B surface antigen, *HBsAb* Hepatitis B surface antibody, *HBeAg* Hepatitis B e antigen, *HBeAb* Hepatitis B e antibody, *HBcAb* Hepatitis B core antibody

The downtrends of the HBeAg, HBeAb, and HBcAb positivity rates in the infants in the two groups are shown in Table 2. The HBeAg positivity rate of infants in group A declined and became negative over 24 months, while the HBeAb and HBcAb positivity rates of infants from birth to month 24 in the two groups declined slowly and remained positive till month 24 for some infants. The HBeAb positivity rate of infants was higher (11.10%) in group B than in group A (0.36%) at month 24. The HBcAb positivity rates of infants in group A vs. group B were 54.80% (952/1737) vs. 53.86% (768/1426) (χ^2^ = 0.248, *P* = 0.618) at month 7–12 and 30.89% (341/1104) vs. 30.69% (271/883) at month 24 (χ^2^ = 0.002, *P* = 0.964), respectively. The positivity rate of HBsAb in the two groups at the two time points was comparable during months 7–12 and month 24 (χ^2^ = 0.052, *P* = 0.819; χ^2^ = 0.317, *P* = 0.573).

## Comparison of HBsAb titers during months 7–12 and in month 24 between the two groups

To determine whether fetal exposure to maternal HBeAg can influence the immune response to neonatal vaccination against HBV, we compared HBsAb levels in infants between the two groups. The median HBsAb titer in group A was 576.9 mIU/ml, which was obviously lower than that in group B (719.7 mIU/ml) at 7–12 months (Z = -3.049, *P* = 0.002). However, the median HBsAb titers in group A and group B in the 24th month were 783.5 mIU/ml and 891.4 mIU/ml and were not significantly different (Z = -0.853, *P* = 0.394). The proportions of medium–high, low, and no response to HBV standard vaccination in neonates with HBeAg positivity at birth were comparable to those in neonates with HBeAg negativity at 7–12 months and 24 months (Table 3).

**Table 3:** The level of HBsAb titer in the two groups during the 7–12 months and in the 24th month

	Infants in A group (*n* = 1737/1104)	Infants in B group (*n* = 1426/883)	Z or X^2^	*P*
during the 7–12 months				
HBsAb titer (mIU/ml)	576.9 (192.8–1000.0)	719.7 (208.1–1000.0)	-3.049	0.002
HBsAb < 10 mIU/ml, n (%)	3(0.2)	2(0.1)	3.629	0.167
HBsAb 10–100 mIU/ml, n (%)	97(5.6)	59(4.1)		
HBsAb ≥ 100 mIU/ml, n (%)	1637(94.2)	1365(95.8)		
in the 24th month				
HBsAb titer (mIU/ml)	783.5 (227.8–1000.0)	891.4 (234.0–1000.0)	-0.853	0.394
HBsAb < 10 mIU/ml, n (%)	12(1.0)	13(1.4)	0.601	0.741
HBsAb 10–100 mIU/ml, n (%)	111(10.1)	87(9.9)		
HbsAb ≥ 100 mIU/ml, n (%)	981(88.9)	783(88.7)		

Abbreviation: *HBsAb* Hepatitis B surface antibody

### Potential risk factors of HBeAg positive at birth in infants

In univariate analysis, mothers with antiviral therapy (OR 5.460, 95% CI 4.151–7.182, *P* < 0.001), higher HBV DNA viral load (OR 1.864, 95% CI 1.734–2.003, *P* < 0.001) and higher HBeAg level (OR 1.003, 95% CI 1.003–1.004, *P* < 0.001) would increase infants’ HBeAg positive rate at birth. However, there was no significant difference in terms of age over 35, vaginal delivery, male gender, gestational week and birth weight. Unconditional multivariate regression analysis was performed after adjusting for the confounding effects of other factors to determine the independent risk factors for HBeAg positivity at birth (Table 4). A significant association of maternal HBV DNA viral load with infants’ positivity of HBeAg at birth was detected (OR 1.260, 95% CI 1.139–1.395, *P* < 0.001). Maternal HBeAg level has an obvious influence on the HBeAg positive rate of infants (*P* < 0.001).

**Table 4:** Factors of HBeAg positive in infants

	Univariate regression analysis			Multivariate logistic analysis		
	OR	95% CI	*P*	OR	95% CI	*P*
With Antiviral therapy	5.460	4.151–7.182	< 0.001	1.398	0.951–2.055	0.089
Maternal HBeAg level	1.003	1.003–1.004	< 0.001	1.003	1.002–1.003	< 0.001
Maternal HBV DNA load	1.864	1.734–2.003	< 0.001	1.260	1.138–1.395	< 0.001
Vaginal delivery	1.199	0.918–1.566	0.183	1.034	0.728–1.467	0.854
Gestational week	1.032	0.938–1.137	0.517	1.029	0.889–1.192	0.699
Birth weight	1.000	1.000–1.000	0.928	1.000	1.000–1.001	0.637
≥ 35 years	0.707	0.449–1.114	0.135	0.725	0.399–1.316	0.291
Male Gender	0.814	0.626–1.059	0.125	0.846	0.597–1.200	0.348
Liver function	0.564	0.413–0.771	< 0.001	0.739	0.494–1.105	0.141

Abbreviations: *CI* Confidential interval, *OR* Odds ratio

### Correlation between HBeAg titers of infants at birth and maternal HBeAg titers before delivery and dynamic changes in infant HBeAg titers during months 7–12

**Fig. 2:**
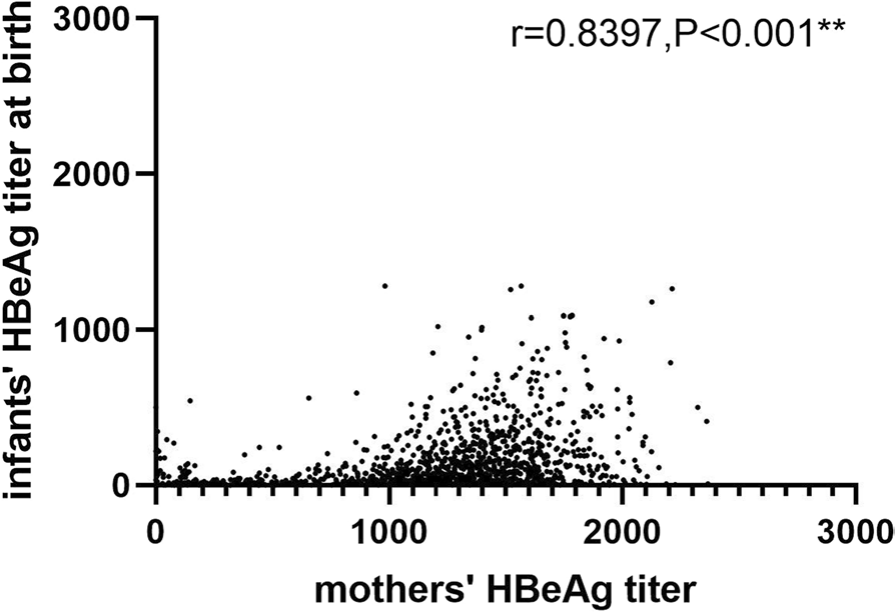
Spearman’s correlation analysis shows the correlation between infants’ HBeAg titer at birth and mothers’ HBeAg titer before delivery

As observed in group A, maternal HBeAg can be transmitted from mothers to infants through the placenta. The infants’ median HBeAg titer at birth was lower than their mothers’ median HBeAg titer (37.9 COI vs. 384.4 COI) (Z = 3,090,541.0, *P* < 0.001). The correlation between the infants’ HBeAg titers at birth and mothers’ HBeAg titers before delivery was analyzed using Spearman’s correlation coefficient, and the results are presented in Fig. 2. The infants’ HBeAg titers were strongly and significantly correlated with their mothers’ HBeAg titers (r = 0.8397, *P* < 0.001). Meanwhile, the median titers of infants at birth and during months 7–12 were 37.9 COI and 0.3 COI, further proving that maternal-derived HBeAg declined rapidly from birth to 7–12 months.

## Discussion

### Principal findings

Hepatitis B virus markers, such as HBeAg, HBeAb, and HBcAb, can be passed from mother to fetus through the placenta. Maternal HBeAg was not persistently present during the 24-month follow-up in the infants and vanished gradually. Maternal HBeAg affected the infant’s immune response to vaccines and temporarily reduced the HBsAb titers in infants in months 7–12; the titers gradually recovered by month 24.

### Strengths of the study

A large scale study was conducted to observe dynamic changes in infant birth HBV markers and to assess the time-varying effects of maternal HBeAg on the immunization of the infants.

### Limitation of the data

First, although no evidence had confirmed that antiviral drugs would affect the infants’ immune function, most HBeAg positive women were treated with antiviral drugs. Differences among the mothers’ infection status may have led to some selection bias. Second, we could not include a control group of infants who received no immunization due to ethical reasons. Moreover, as the manuscript was based on a retrospective study, we could not conduct related immunological examinations on the same cohort at the two time points, which would require further investigation in the future. Third, infants were not followed up for a longer period than 24 months; this would have been especially helpful for infants who were persistently positive for HBcAb and HBeAb till month 24.

### Interpretation

HBV can be transmitted from mothers to infants for its ability to pass through the placental barrier. It is noted that HBeAg of infants at birth all came from their mothers [13, 14]. On this basis, we conducted a study and compared the HBeAg positive rate of infants born to HBeAg positive mothers and discovered potential factors. The overall HBeAg positive rate of infants in the current study was 90.2%. It is noted that mothers with antiviral therapy would increase infants’ HBeAg positive rate at birth in our univariate analysis. While the level of maternal HBeAg and HBV DNA load was found to be risk factors for infants’ HBeAg positive rate after univariate and multivariate regression analysis. It is universally accepted that women with high viral load and HBeAg positivity are recommended to receive antiviral therapy during gestational period to prevent MTCT. That is to say, antiviral therapy and high viral load or HBeAg positivity are in the same trend. Synergistic effect was eliminated after adjusting for the confounding effects in multivariate analysis. Our research results are consistent with those of Wan-Hsin Wen et al. [15]. HBeAg is a soluble antigen with a small molecular weight. It is a derivative of HBcAb and not a component of the HBV particle. HBeAg is secreted into the host circulation during viral replication and is related to the impairment of both host innate and adaptive immune responses in persistent HBV infection of adult [16]. HBeAg can be used as an index of viral replication, infectivity, inflammation, and disease severity as well as response to antiviral therapy in adults. Women with chronic HBV infection at child-bearing age, especially with HBeAg positivity, require intensive attention and care because changes in their immune system during pregnancy may worsen their HBV infection. Moreover, HBV may also be transmitted by these women to their offspring. Although combined immunoprophylaxis with vaccines and HBIG is currently the most effective way to prevent MTCT of HBV, it can’t completely prevent HBV transmission, [17, 18] especially in cases of mothers with both HBsAg and HBeAg positivity. HBeAg can be used as an alternative for MTCT prediction if HBV DNA detection is unavailable [1, 19].

The HBeAg titer of infants at birth was significantly lower than that of their mothers in this study. The ability of HBeAg to pass through the placenta may depend upon its molecular size or HBV particle morphology [20]. The placental barrier could prevent some maternal HBeAg from being transmitted to offspring. Damage of the placental barrier causes fetal exposure to HBV and HBeAg and might therefore be one of the reasons for MTCT. The mechanism underlying the effect of maternal HBeAg on infant immunization has remained controversial, and there has been a lack of detailed investigations. Therefore, we performed a study using a large sample size to assess the influence of maternal HBeAg on the immunization effect in infants born to mothers infected with HBV and the dynamic changes in these infants’ birth HBV markers over a long follow-up period. We suppose that HBeAg derived from mothers are not active and do not cause infection in infants. Although the HBeAg ( +) positivity rate of children aged 7–12 months in Group A is 12.8%, the HBeAg titer is at low level in most infants. All HBeAg switched to negative at 24 months and did not affect the infants’ response to vaccine. Furthermore, the maternal derived HBeAg did not cause infection in infants in our cohort. That is why the HBsAg positivity rate is 0% at 7–12 months.

We found that the HBsAb titer of infants in group A at 7–12 months was significantly lower than that in group B (576.9(192.8–1000) vs. 719.7(208.1–1000), *P* = 0.002) from a large sample size. However, there was no significant difference in HBsAb titers at month 24 between group A and group B. Our results showed that maternal HBeAg might lead to temporary immune inhibition in infants during months 7–12, but this inhibitory effect gradually subsided by month 24. It is universally accepted that neonates born to HBsAg positive mothers have immune tolerance to HBV proteins due to exposure in utero. HBeAg is a Th-cell dependent antigen, while HBcAg is a Th-cell-independent antigen [21, 22]. David R et al. [23] reported that in utero exposure to HBeAg leads to Th-cell tolerance, preventing response to HBeAg and HBcAg in transgenic mice models. The cell tolerance may be responsible for chronic HBV infection acquired perinatally and, thereby, poor prognosis. This could explain the lower HBsAb titer in group A in months 7–12. All infants in our investigation received HBV vaccine and HBIG immunization to aid the establishment of their immune systems. We propose that Th-cell tolerance subsides with the maturation of the infant’s immune system upon stimulation by scheduled immunization. This may be why the HBsAb titers at month 24 showed no difference between group A and group B.

Our findings were different from those of Huang et al. They compared HBsAb levels in months 7–12 between 124 infants with HBeAg positive cord blood and 141 infants with HBeAg negative cord blood. The HBsAb levels and their dynamic changes were also compared between the two groups. In their study, fetal HBeAg exposure did not inhibit the antibody response to neonatal hepatitis B vaccination and induce infant immune tolerance to HBV in months 7–12. The differences between the findings of our investigation and that of Huang et al. may be due to differences in sample size, sample source, and follow-up period. Furthermore, the titer levels of HBsAb between group A and group B showed no difference in 24 in our study, similar to the findings of Huang et al. Therefore, we propose the hypothesis that fetal exposure to HBeAg only has a negative effect on infant immunization within a few months after birth.

During our follow-up, it was also noted that infants with an HBsAb titer over 100 mIU/ml (medium–high immunization response) switched to no or low immunization response. Therefore, we decided to focus on the dynamic changes in HBsAb titers among infants after birth, especially in the case of maternal HBeAg positive infants. Prolonged follow-up of the HBsAb titers of infants was required to assess the protective effects of immunization. Meanwhile, the infants’ HBV markers presented different modes of expression at birth and depended largely on the expression modes of the mothers; these findings were similar to those of other studies. HBeAg, HBeAb, and HBcAb could pass through the placenta and their positivity rates displayed a downtrend during our follow-up. The positivity rates of maternal-derived HBeAg in group A declined rapidly over months 7–12 and all switched to negative by month 24. This trend could explain the changes in the infants’ HBsAb titers and immune response levels, further verifying the potential immune tolerance or inhibition effect of maternal HBeAg.

Maternal HBcAb and HBeAb in infants could last for a longer period (till month 24) than other HBV markers in our study. This result was not consistent with those of other studies, which reported that HBcAb and HBeAb disappeared before month 24 [24]. In recent years, some researchers have proposed the concept of occult hepatitis B infection (OBI), [25, 26] which is characterized by HBsAg negativity, natural immunity (with HBcAb positivity), and low-level viremia within the liver, serum, and extrahepatic reservoirs (peripheral blood mononuclear cells or lymphoid system) [27]. For infants with persistent HBcAb positivity till month 24, we couldn’t exclude the possibility of OBI. Therefore, we recommend paying more attention to such infants and observing dynamic changes in their HBV markers until they disappear. Most infants could produce effective HBsAb even with persistent HBeAb and/or HBcAb positivity till month 24 in our study. These findings demonstrate that HBV markers received passively from mothers are not active and do not involve in HBV replication in infants’ livers, as opposed to HBV markers in adults, which are produced by chronic hepatitis B virus infection.

## Conclusions

The present study revealed that birth HBV markers of infants varied according to the expression modes and titers of their mothers’ HBV markers. Maternal HBeAg did affect the infants’ immune response to vaccination and reduce the anti-response at 7-12th month temporarily, but these influences were negligible by 24th months after birth, which proved that the maternal HBeAg would not induce immune tolerance of infants from a long-term perspective. Maternal HBcAb and HBeAb in infants could last for a longer period (till month 24) than other HBV markers in our study, which could not exclude the possibility of OBI. Therefore, follow-up of successfully vaccinated infants born to mothers with HBsAg should therefore be continued till adolescence or early adulthood, especially for infants in close household contact with HBV-infected individuals.

## Data Availability

The data were extracted for analysis using our electronic database which was built in previous research and is not available to the public, but are available from the corresponding author upon reasonable request.

## Abbreviation

HBsAg: Hepatitis B surface antigen
HBsAb: Hepatitis B surface antibody
HBeAb: Hepatitis B e antibody
HBcAb: Hepatitis B core antibody
HBV: Hepatitis B virus
MTCT: Mother-to-child transmission
HBeAg: Hepatitis B e antigen
HBcAg: Hepatitis B core antigen
